# CPI-17 drives oncogenic Ras signaling in human melanomas via Ezrin-Radixin-Moesin family proteins

**DOI:** 10.18632/oncotarget.12919

**Published:** 2016-10-26

**Authors:** Lars Björn Riecken, Ansgar Zoch, Ulrike Wiehl, Sabine Reichert, Ingmar Scholl, Yan Cui, Mirjana Ziemer, Ulf Anderegg, Christian Hagel, Helen Morrison

**Affiliations:** ^1^ Leibniz Institute on Aging, Fritz Lipmann Institute, Jena, Germany; ^2^ Department of Cell and Developmental Biology, University College London, London, United Kingdom; ^3^ Klinik und Poliklinik für Dermatologie, Venerologie und Allergologie, Universität Leipzig, Leipzig, Germany; ^4^ Department of Neuropathology, University Medical Center Hamburg-Eppendorf, Hamburg, Germany

**Keywords:** CPI-17, ERM, Ras, cancer, melanoma

## Abstract

Hyperactive Ras signaling has strong oncogenic effects causing several different forms of cancer. Hyperactivity is frequently induced by mutations within Ras itself, which account for up to 30% of all human cancers. In addition, hyperactive Ras signaling can also be triggered independent of Ras by either mutation or by misexpression of various upstream regulators and immediate downstream effectors. We have previously reported that C-kinase potentiated protein phosphatase-1 inhibitor of 17 kDa (CPI-17) can drive Ras activity and promote tumorigenic transformation by inhibition of the tumor suppressor Merlin. We now describe an additional element of this oncogenic mechanism in the form of the ezrin-radixin-moesin (ERM) protein family, which exhibits opposing roles in Ras activity control. Thus, CPI-17 drives Ras activity and tumorigenesis in a two-fold way; inactivation of the tumor suppressor merlin and activation of the growth promoting ERM family. The *in vivo* significance of this oncogenic switch is highlighted by demonstrating CPI-17's involvement in human melanoma pathogenesis.

## INTRODUCTION

The small GTPase Ras is a central signaling component that translates a multitude of extracellular stimuli into well-defined cellular processes - including proliferation, migration and differentiation [1]. Aberrant Ras activity has strong oncogenic effects, causing several different forms of cancer [1–3]. Tumor cells exhibiting hyperactive Ras signaling are independent from extracellular mitogenic input, thus showing uncontrolled cellular growth and proliferation [1, 3]. Hyperactive Ras signaling is frequently induced by mutation of Ras itself [1–3]; Ras mutations account for up to 30% of all human cancers [2]. However, hyperactive Ras signaling can also be induced independent of Ras mutations; either through mutation or through misexpression of various upstream regulators and immediate downstream effectors [1, 2, 4, 5].

Previously, we have reported that C-kinase potentiated protein phosphatase-1 inhibitor of 17 kDa (CPI-17) can drive Ras activity and promote tumorigenic transformation by inhibiting the tumor suppressor Merlin [6]. CPI-17 directly controls Merlin activity by regulating its phosphorylation status via the myosin phosphatase MYPT1-PP1δ. We now describe an additional element of this oncogenic mechanism in the form of the ezrin-radixin-moesin (ERM) protein family. Although closely related, Merlin and the ERM proteins are regulated in an opposing manner, i.e. C-terminal phosphorylation inactivates Merlin but activates ERM proteins [7, 8]. Moreover, Merlin and ERM proteins fulfil opposing roles in Ras activity control; while Merlin restricts Ras activity [5], ERM proteins are essential for proper Ras activation [9–11]. Here we show that ERM proteins, like Merlin, are regulated by a CPI-17 - MYPT1-PP1δ switch, which results in hyperactive Ras signaling and tumorigenic growth. Thus, we report CPI-17 as a potent oncogene driving Ras activity and tumorigenesis in a two-fold way - inactivation of the tumor suppressor Merlin and activation of the growth promoting ERM family. Consequently, we find that misexpression of CPI-17 in a cell line physiologically devoid of CPI-17 suffices to drive Ras activity as well as cellular transformation.

Moreover, we have previously reported misexpression of CPI-17 in different cancer cell lines, including Recurrent Primary Malignant Melanoma cells (RPM-MC) [6]. We now provide further evidence demonstrating CPI-17's involvement in human melanoma pathogenesis by reporting frequent misexpression in primary patient-derived melanoma tissue and cell line samples. Conversely, depletion of CPI-17 in patient-derived melanoma cell lines decreases proliferation, cellular transformation and hyperactive Ras signaling. Strikingly, we identified CPI-17 misexpression particularly in those samples devoid of oncogenic BRAF or NRAS mutations. Thus, we propose CPI-17 as a potent oncogene providing an alternative mechanism to drive oncogenic Ras signaling and tumorigenesis.

## RESULTS

**Figure 1:**
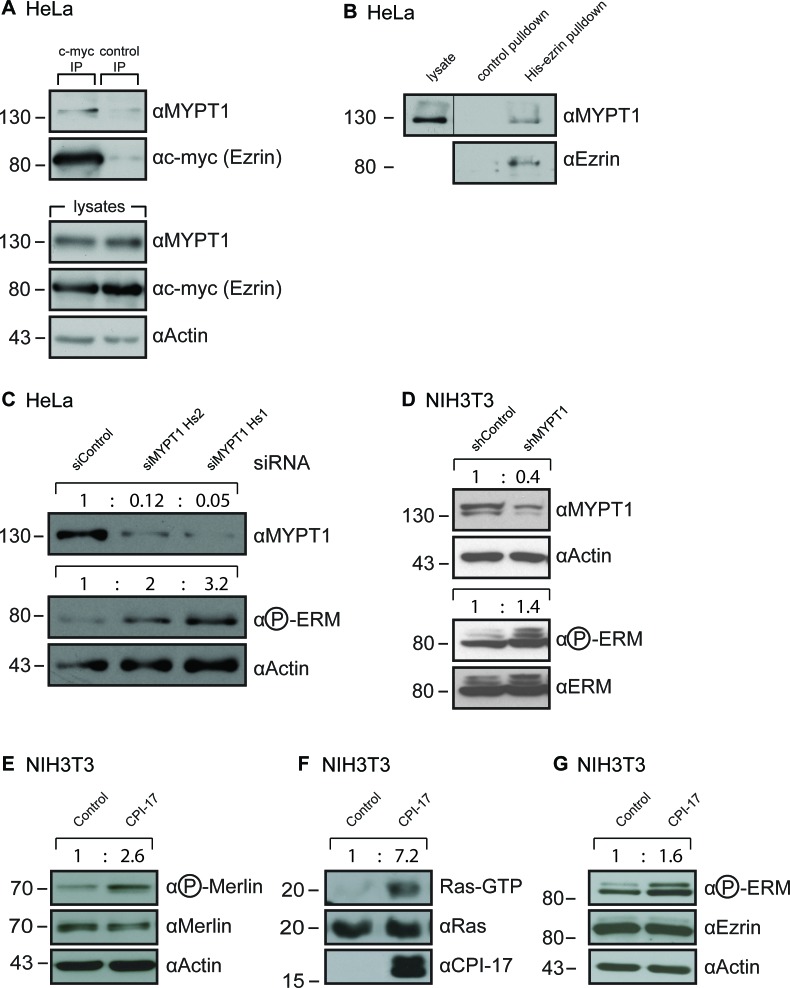
MYPT1 regulates ERM phosphorylation (**A**-**B**) MYPT1 readily co-precipitates with ezrin from HeLa cells. A: Ezrin was immunoprecipitated from HeLa cells expressing a myc-tagged ezrin. B: MYPT1 was precipitated from HeLa cells using recombinant purified His-tagged ezrin coupled to Ni-Sepharose. (**C**) Depletion of MYPT1 in HeLa cells increases ERM phosphorylation. Two independent siRNAs targeting different regions of the MYPT1 mRNA were used. (**D**) Depletion of MYPT1 in NIH3T3 cells increases ERM phosphorylation. Cells stably express shRNA targeting either Luciferase (shControl) or MYPT1 mRNA (shMYPT1). (**E**-**G**) Overexpression of the MYPT1 inhibitor CPI-17 in NIH3T3 cells increases (**E**) inhibitory phosphorylation of merlin (**F**) induction of the small GTPase Ras and (**G**) activating phosphorylation of ERM proteins. Quantification of band size performed by ImageJ, normalization to Actin (C-E), total Ras (F) or ezrin / ERM (D, G) levels.

We previously reported that MYPT1 binds directly to Merlin to mediate its dephosphorylation [6]. Others have reported that MYPT1 can also bind directly to the closely related ERM family members ezrin and moesin *in vitro* [12]. Binding to moesin was additionally confirmed within a cellular context using MDCK cells [12]. For this reason, we hypothesized that ezrin, too, is bound and regulated by MYPT1 *in vivo*. Indeed, interaction of ezrin with MYPT1 was readily detected by co-immunoprecipitation from HeLa cells transiently expressing a myc-tagged ezrin protein (Figure 1A). This result was further validated by affinity precipitation, using recombinantly expressed His-tagged ezrin protein on HeLa lysates (Figure 1B). In order to test whether MYPT1 also mediates ezrin's dephosphorylation, we downregulated MYPT1 expression in HeLa cells using two independent siRNAs. Loss of MYPT1 expression consistently yielded a strong increase in ezrin phosphorylation, correlating noticeably with the efficiency of MYTP1 depletion (Figure 1C).MYPT1-dependent ezrin dephosphorylation was also validated by downregulation of MYPT1 expression in NIH3T3 mouse fibroblasts (Figure 1D), using a lentiviral shRNA approach. Thus, in addition to Merlin, MYPT1-PP1δ also appears to mediate dephosphorylation of the closely related ezrin protein and, by extension, likely the whole ERM protein family.

Previously, we have reported on the tumorigenic potential of the MYPT1-PP1δ inhibitor CPI-17, due to increased phosphorylation and, thus, inactivation of the tumor suppressor Merlin [6]. We validated this finding by generating NIH3T3 fibroblasts stably expressing CPI-17 (Figure 1E-1G). As per previous results, overexpression of CPI-17 led to a strong increase of inhibitory Merlin phosphorylation (Figure 1E) and resulted in robust activation of the small GTPase Ras (Figure 1F). We also observed a marked increase in activating phosphorylation of ERM proteins (Figure 1G) - counter players of Merlin in Ras activity control [11]. Therefore, the CPI-17 - MYPT1-PP1δ axis may employ dual mechanisms to drive Ras pathway activity; inactivation of the tumor suppressor Merlin to release inhibition of Ras activity, and activation of the growth promoting ERM protein family to increase Ras activation.

**Figure 2:**
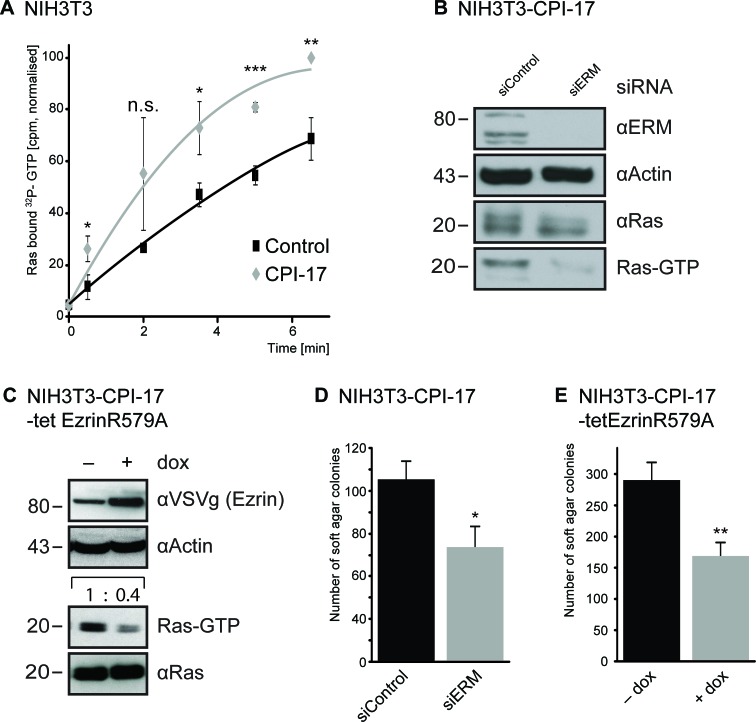
CPI-17 drives Ras activation and cellular transformation via ERM proteins (**A**) GEF activity measured by increase of ^32^P-labeled Ras in streptolysin O (SLO)-permeabilized NIH3T3 cells treated with [α^32^P]-GTP and 10 ng/ml PDGF. (**B**.-**E**) Depletion of ERM protein functions by either siRNA transfection (B, D) or over expression of the dominant negative ezrinR579A (C, E) counters CPI-17 induced B-C: Ras activity and D-E: soft agar colony formation of NIH3T3 fibroblasts. Statistical analysis performed by two-tailed t-test (A: *p* = 0.02 / 0.15 / 0.02 / 0.0004 / 0.02 C: *p* < 0.05 E: *p* < 0.005) pre-requisites tested by F-test (A: *p* = 0.97 / 0.001 / 0.32 / 0.4 / <0.001 C: *p* > 0.89, E: *p* > 0.81).

To test this possibility, we measured [α^32^P]-GTP loading of Ras *in vivo*, using streptolysin O (SLO)-permeated cells (Figure 2A). Radioactive labeling of the α-phosphate allows measurement of Ras-GTP loading (facilitated by ERM proteins [10]), independent of simultaneously occurring GTP to GDP hydrolysis (facilitated by Merlin [5]). This enables direct measuring of Ras activity induction (see Material and Methods for details). NIH3T3 cells expressing CPI-17 showed significantly faster ^32^P-labeling of Ras; detectable after 30 seconds and resulting in strongly increased ^32^P-labeled Ras at all time points measured (Figure 2A). Hence, CPI-17 not only reduces Ras inhibition due to inactivation of Merlin, but also drives induction of Ras activation - presumably due to activation of ERM proteins, which we previously reported to actively facilitate GTP-loading and, thus, activation of Ras [10]. In support of this, depletion of ERM proteins in CPI-17 expressing NIH3T3 cells strongly reduced active GTP-Ras levels (Figure 2B). Likewise, expression of a dominant negative ezrin mutant (ezrinR579A [13]) strongly reduced GTP-Ras levels (Figure 2C); further confirming the requirement of functional, active ERM proteins for CPI-17 driven Ras activation. Subsequently, we tested whether CPI-17 employs ERM proteins not only to induce Ras activity, but also to drive cellular transformation. To this end, we deprived CPI-17 expressing NIH3T3 cells of ERM protein functions by either siRNA-mediated ERM depletion (Figure 2D), or overexpression of the dominant negative mutant ezrinR579A (Figure 2E). Downregulation of ERM activity by either method provoked a significant decrease in the number of colonies formed in soft agar - confirming ERM proteins as vital contributors of CPI-17's tumorigenic potential.

**Figure 3:**
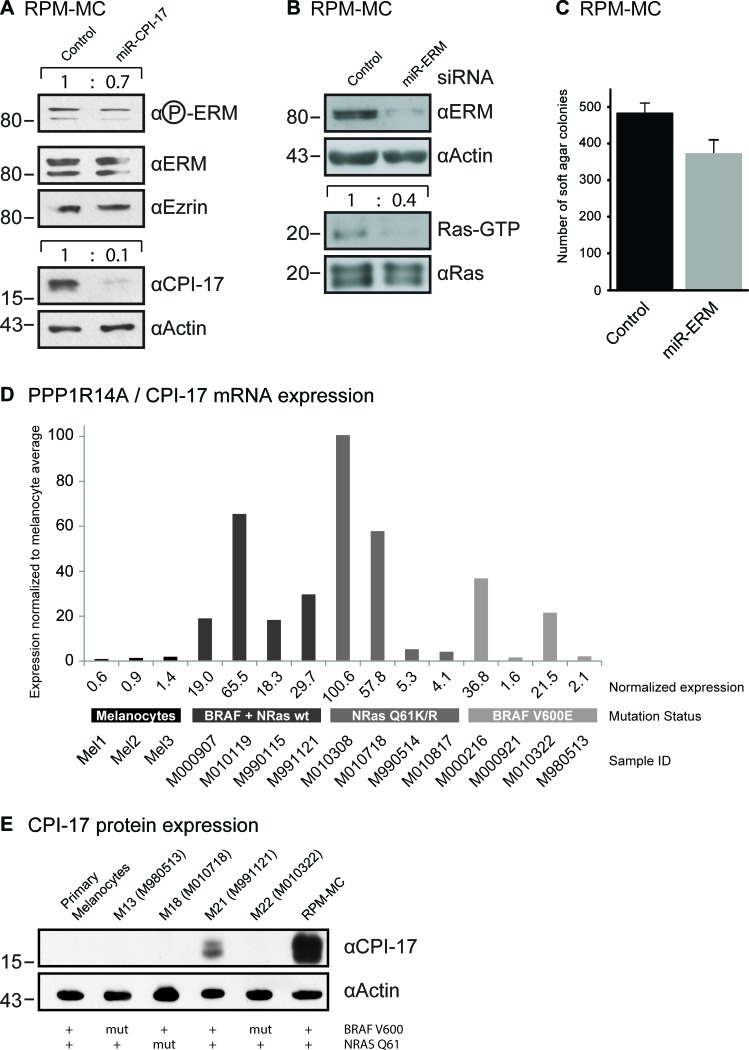
CPI-17 drives oncogenesis in RPM-MC cells and is frequently misexpressed in human melanoma samples (**A**) Depletion of CPI-17 decreases ERM phosphorylation in stably transduced RPM-MC cells. (**B.**-**C**) Depletion of ERM proteins in RPM-MC cells by lentivirally delivered miRNA inhibits B: Ras activity and C: soft agar colony formation. (**D**) Microarray analysis of CPI-17 mRNA expression in different human melanoma samples and primary human melanocytes. Overexpression is obvious in 10 of 12 samples overall and 4 of 4 samples devoid of N-Ras and BRAF mutations. Microarray data was previously reported in [16] and accessed via NCBI GEO database [29]. Quantification of western blots performed by ImageJ, normalization to total Ras levels. E: Misexpression of CPI-17 protein in patient-derived melanoma cell lines, cells derived from samples used for (D). Misexpression is detectable only in BRAF V600, NRAS Q61 wild type cells and absent from primary human melanocytes.

Physiologically, expression of CPI-17 is restricted to few select cell types, primarily smooth muscle cells [14, 15], but absent from most other cell types. In contrast, we previously found CPI-17 misexpressed in several different cancer cell lines [6], including Recurrent Primary Malignant Melanoma Cells (RPM-MC). Downregulation of CPI-17 in RPM-MC cells strongly reduced hyperactive Ras levels and soft agar colony formation [6], suggesting CPI-17's involvement in melanoma tumorigenesis. As previously observed in transformed NIH3T3 fibroblasts (Figure 2), ERM proteins appeared to be vital contributors to CPI-17's tumorigenic potential in melanoma cells. Depletion of CPI-17 in RPM-MC cells lowered ERM phosphorylation (Figure 3A), while depletion of ERM proteins alleviated CPI-17's effect on Ras activation (Figure 3B) and soft agar colony formation (Figure 3C); confirming ERM proteins as important components of CPI-17's oncogenic role in melanoma cells.

**Figure 4:**
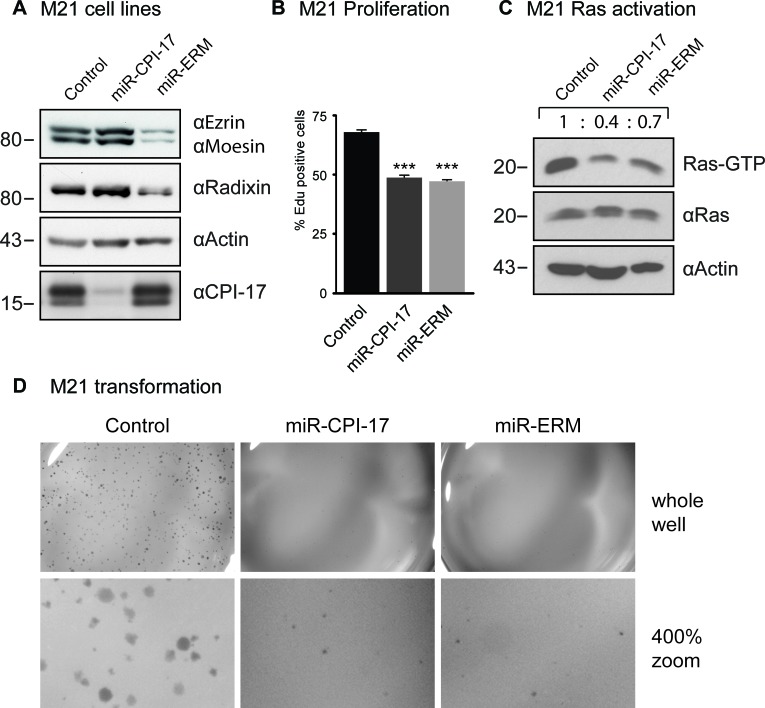
CPI-17 drives oncogenesis in patient-derived M21 melanoma cells (**A**) Stable knockdown cell lines were generated by transducing artificial miRNA targeted against CPI-17 or ezrin, radixin and moesin (ERM) respectively. (**B.**-**D**) Depletion of either CPI-17 or ERM proteins in M21 cells decreases B: proliferation C: Ras activity and D: soft agar colony formation. Proliferation data derived from six independent wells with > 1700 cells detected per well. Soft agar images are representative for three (CPI-17) or two (ERM) experiments with triplicates each (Supplementary Figure S3). Statistical analysis performed by two-tailed t-test (*t*-test: *p* < 5 × 10^−7^), pre-requisites tested by F-test (*p* = 0.89 and *p* = 0.49, respectively).

To assess the significance and prevalence of CPI-17's tumorigenic role in human melanoma pathogenesis, we analyzed CPI-17 expression in several melanoma-derived cell samples, as well as in normal human melanocytes previously reported in the Zürich dataset [16] (Figure 3D). CPI-17 mRNA was overexpressed in the majority of samples - with two thirds (8 of 12) showing at least 10-fold increased expression. CPI-17 was consistently and highly overexpressed (4 of 4) in melanoma samples containing neither oncogenic BRAF V600E nor oncogenic NRas Q61K/R mutations; suggesting that Ras activity may primarily be driven via the CPI-17-ERM pathway in these cells. This assumption is supported by analysis of CPI-17 expression and function in melanoma cell lines derived from the data set (Figure 3E, Figure 4). Misexpression of CPI-17 protein was specifically detected in melanoma cell lines wildtype for both the BRAF V600 and NRAS Q61 locus (Figure 3E; see Supplementary Figure S1 for sequencing results of RPM-MC cells) and absent in primary melanocytes. Expression of the CPI-17 effectors ezrin, radixin and moesin appeared unchanged and no loss of Merlin expression was detected (Supplementary Figure S2). In agreement with our previous results obtained in RPM-MC cells, depletion of either CPI-17 or ERM expression (Figure 4A) in M21 melanoma cells led to a significant decrease in proliferation (Figure 4B), Ras activation (Figure 4C), and strongly inhibited cellular transformation (Figure 4D, Supplementary Figure S3); further demonstrating CPI-17's tumorigenic potential involving ERM proteins in melanoma cells.

**Figure 5:**
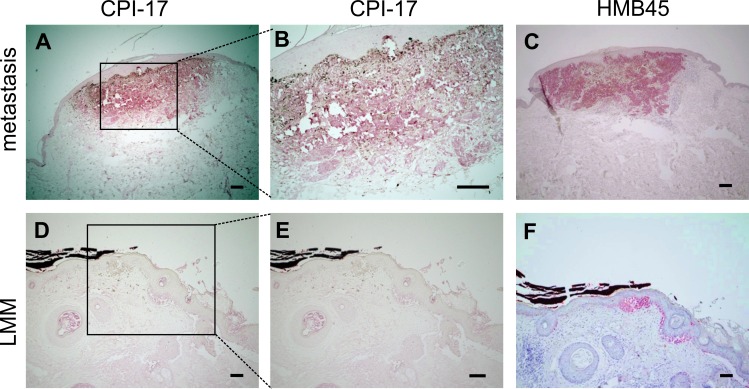
CPI-17 expression in human melanoma tissue samples correlates with HMB45 reactivity (**A.**-**F**) Immunohistochemical CPI 17 and HMB45 staining (in red) of formalin-fixed paraffin-embedded melanoma samples. A-B: Strong staining of CPI-17 is observed in a melanoma metastasis and colocalizes with C: HMB45 staining specific for melanocytic tumors (C). D-E: Weak staining in a lentigo maligna melanoma (LMM) indicates correlation of staining intensities of CPI-17 and F: the HMB45 tumor marker Scale bars: A, C, D, F: 200 μm and B, E: 100 μm.

Further investigating the prevalence of CPI-17 misexpression in human melanomas, we analyzed several tissue samples derived from patient material. Around 70% of metastatic and nodular malignant melanomas (NMM) - as well as 50% of superficial spreading melanomas (SSM) - showed strong CPI-17 expression, compared to only 20% of benign nevi samples (Table 1). Moreover, delineation of the melanocytic tumor area by the HMB45 antibody showed clear localization of CPI-17 expression to the tumor area, while the surrounding healthy tissue was negative for CPI-17 expression (representative images in Figure 5A-5F). Together, this data promotes CPI-17 as a key regulator of a novel oncogenic signaling cascade driving Ras activity and cellular transformation of melanoma cells.

**Table 1:** Melanocytic tumours positive for CPI 17 expression

		CPI-17						HMB-45			
Tumor samples		positive (total)		positive (strong)		negative		positive		negative	
**SSM**	10	8	(80%)	5	(50%)	2	(20%)	8	(80%)	2	(20%)
**ALM**	10	8	(80%)	4	(40%)	2	(20%)	9	(90%)	1	(10%)
**NMM**	10	10	(100%)	7	(70%)	0	(0%)	9	(90%)	1	(10%)
**LMM**	9	7	(78%)	3	(33%)	2	(22%)	5	(56%)	4	(44%)
**Metastatis**	10	10	(100%)	7	(70%)	0	(0%)	9	(90%)	1	(10%)
**Nevi (NCN)**	10	6	(60%)	2	(20%)	4	(40%)	4	(40%)	6	(60%)
**All tumours**	59	49	(83%)	28	(47%)	10	(17%)	44	(75%)	15	(25%)

SSM: superficial spreading melanoma, ALM: Acral lentiginous melanoma, NMM: nodular malignant melanoma, LMM: Lentigo maligna melanoma, NCN: Nevocellular Nevus

## DISCUSSION

**Figure 6:**
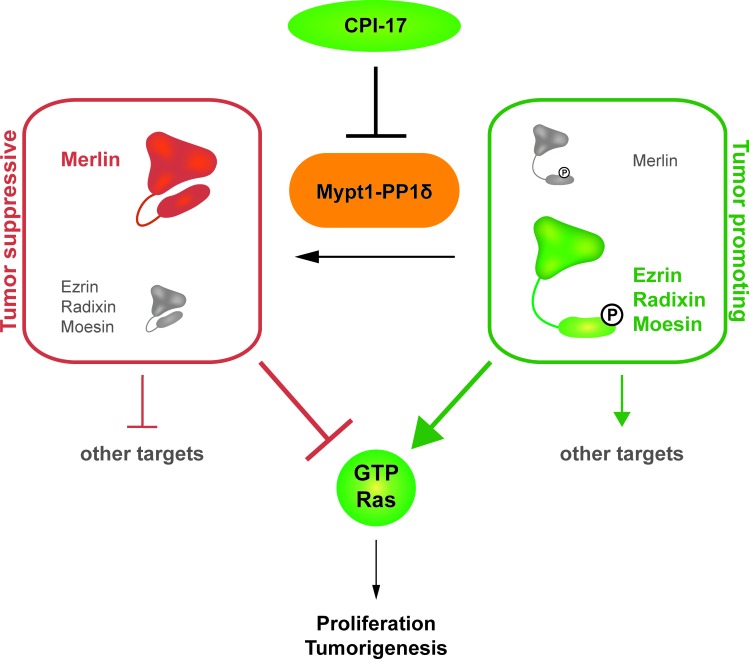
CPI-17 is a key regulator of a novel oncogenic signaling cascade The CPI-17 - Myp1-PP1δ axis constitutes a regulatory switch between a phosphorylated, growth promoting and a dephosphorylated, growth inhibiting state of the physiological counter players Merlin and the ERM protein family. Subsequently, phosphorylated ERM proteins induce Ras activity while dephosphorylated Merlin inhibits Ras. Additional downstream targets of both Merlin and the ERM proteins suggest that CPI-17 indirectly regulates a broad spectrum of downstream signaling cascades.

This study identified ERM proteins as downstream targets of a regulatory CPI-17­-MYPT1-PP1δ switch controlling ERM phosphorylation and thus activation (Figure 1, Figure 3A, Figure 6). Both depletion of MPYT1 and overexpression of its inhibitor CPI-17 were sufficient to strongly induce ERM phosphorylation (Figure 1C, 1D, 1G). Ezrin readily co-precipitated MYPT1 from HeLa lysates (Figure 1A-1B), while moesin had previously been reported to interact with MYPT1 in MDCK cells [12]. Moreover, both ezrin's and moesin's FERM domain were shown to directly bind MYPT1 *in vitro* [12]. Combined, these results indicate that MYPT1 interaction capabilities may be a shared feature of the entire ERM protein family, including radixin.

The ERM protein family - ezrin in particular - has been repeatedly linked to tumorigenesis and metastatic progression (reviewed in [17]). High expression of ezrin in tumors consistently correlates with a bad prognosis, although the molecular mechanism often remains poorly understood. We have previously demonstrated that ezrin directly participates in the activation of the small GTPase Ras [10, 11]. Hyperactive Ras signaling is one of the most frequent drivers of tumorigenesis - up to 30% of all human tumors harbor Ras mutations [2]. In addition, mutation of Ras regulatory proteins rather than Ras itself offer additional mechanisms to drive oncogenic activity [1]. Our present study reports misexpression of CPI-17 as an alternative mechanism to induce and/or potentiate oncogenic Ras pathway signaling by means of the ERM protein family.

We previously reported CPI-17 as a driver of cellular transformation and tumorigenesis through inactivation of the tumor suppressor Merlin. In contrast to ERM proteins, Merlin inhibits Ras pathway signaling by promoting inactivation of Ras. Thus, CPI-17's oncogenic potential appears to be at least two-fold - inactivation of the tumor suppressor Merlin and activation of the growth promoting ERM protein family, to drive Ras pathway activity and tumorigenesis (Figure 6). In addition, as recent studies have identified both Merlin and ERM proteins as central signaling hubs (reviewed in [17–19]), CPI-17 likely deregulates additional oncogenic pathways dependent on either protein (Figure 6), e.g. members of the Rho family GTPases such as Rac1 [5, 20, 21], RhoA [22–24], or the hippo pathway [25].

We note that CPI-17-induced cellular transformation was only partially blocked by ERM depletion in RPM-MC cells, suggesting the involvement of additional CPI-17-dependent processes. For one, depletion of ERM proteins likely did not alleviate CPI-17-dependent inhibition of the tumor suppressor Merlin, thus allowing residual Ras activity to continue driving transformation. In support of this, inhibition of cellular transformation upon depletion of ERM proteins appeared to be stronger in the M21 cell line (Figure 4D, Supplementary Figure S3), which exhibit lower Merlin protein levels than the RPM-MC cell line (Supplementary Figure S2). For another, while we have established ERM proteins (and Merlin [6]) as important downstream targets of the CPI-17 - MYPT1-PP1δ axis, both CPI-17 and MYPT1-PP1δ may regulate additional downstream targets. Thus, other pathways independent of Merlin and/or ERM proteins may likewise be misregulated and contribute to cellular transformation and tumorigenesis (Figure 6) - further potentiating CPI-17's oncogenicity.

In the present study we have identified CPI-17 as a potent oncogene that is frequently misexpressed in different types of melanocytic tumors (Figure 3D-3E, Figure 5, Table 1). Others have reported CPI-17 to be frequently misexpressed in human mesothelioma [26] while we have reported misexpression in several additional cell lines derived from pancreatic, hepatic, ovarian or cervical carcinomas [6] - suggesting CPI-17's involvement also in other tumor types. In summary, we propose CPI-17 to be a key regulator of an oncogenic signaling cascade driving tumorigenesis in multiple different ways. Therefore, CPI-17 should be regarded as a strong tumor promoter and considered as a therapeutic target for individualized small molecule inhibitor development.

## MATERIALS AND METHODS

### Material/reagents and kits

Recombinant human platelet-derived growth factor (PDGF) BB was obtained from Biomol, G418 from Life Technologies, Doxycycline (dox) from Sigma-Aldrich, Lubrol 17A17 from Uniqema, protein A agarose from Dianova, Ni-Sepharose 6 Fast Flow from GE Healthcare, Active Ras Pulldown and Detection Kit from Thermo Fisher Scientific.

### Antibodies

Following antibodies were used for western blotting: Actin (I-19), Ezrin (C-19), Radixin (C-15), Moesin (C-15), Merlin (B-12), MYPT1 (H-130), c-myc (9E10) from Santa Cruz Biotechnology; CPI-17 (E305) from Epitomics; Ezrin (3C12) from Neomarkers; Ezrin (3145), Radixin (C4G7), phospho-ERM (3149) from Cell Signaling Technology; Moesin (EPR2429(2)) from Abcam; phospho-NF2 S518 from US Biologicals; VSVg from Roche. For Ras, either the antibody from the Active Ras Pulldown and Detection Kit from Thermo Fisher Scientific was used, or Ras (EP1125Y) from Abcam (Figure 4C). For immunoblots of ERM (unless otherwise stated), a mixture of Ezrin (C-19), Radixin (C-15) and Moesin (C-15) antibody was used; for single Ezrin blots, the 3C12 antibody was used. Ezrin (3145), Radixin (C4G7) and Moesin (EPR2429(2)) were used for Figures 1D, 3A, 4A and Supplementary Figure S2. Staining of paraffin sections was performed using anti-CPI-17 from Millipore and HMB45 from DAKO. Ras antibody Y13-259 (Thermo Fisher Scientific) was used for the GEF assay.

### Plasmids

Human ezrin wildtype with C-terminal myc-tag was cloned in pcDNA3.1 (Invitrogen); human Ezrin mutant R579A with C-terminal VSVg-tag was cloned in pUHD10.3 rtTA responsive cloning vector (EcoR1/Xba1) (Invitrogen). Human CPI-17 wildtype containing an N-terminal FLAG-tag was cloned in pcDNA3.1-V5-His-TOPO (Invitrogen). For protein purification, human ezrin wildtype with N-terminal His-tag was cloned in pET15TEV expression vector [10].

### siRNAs, shRNAs and miRNAs

Down-regulation of protein expression was achieved as indicated either by transient transfection of short interfering RNA (siRNA), or stable transduction with lentiviral particles coding for short-hairpin RNA (shRNA) under the U6 promoter, or artificial micro-RNA (miRNA) under a CMV promoter. siRNAs were purchased from Ambion (only the sense strand is reported): ezrin (mouse): 5′-GCCGUAUGUAGACAAUAAAGG-3′; radixin (mouse): 5′-GCACCUCGUCUGAGAAUCAAU-3′; moesin (mouse): 5′- GCAAGCCUGACACCAUUGAGG-3′, ezrin (human): 5′-CCCCAAAGAUUGGCUUUCC-3′, radixin (human): 5′-GCAGUUGGAAAGGGCACAA-; moesin (human): 5′-AAAAGCCCCGGACUUCGUC-; MYPT1 (human, Hs1): 5′-GCAGGCUAUGAUGUUAAUA-3′ and MYPT1 (human, Hs2): 5′-GAGACAAGAAAGAUUUGCU-3′; GL2 Luciferase siRNA was used as control: 5′-CGUACGCGGAAUACUUCGA- 3′. For siRNA-mediated triple knockdowns the amount of control siRNA equaled the sum of all three targeting siRNAs. Knock-down efficiency was tested after 2-3 days. Following shRNA target sequences were used: Control (Firefly Luciferase): 5′- GATATGGGCTCACTGAGACT-3′; MYPT1 (mouse): 5′-GGAACTAACGGATCTAAAGT-3′. Following miRNA target sequences were used: ezrin (human): 5′-AACCCCAAAGATTGGCTTTCCT -3′, radixin (human): 5′-AAGCAGTTGGAAAGGGCACAAT-3′; moesin (human): 5′-AGATCGAGGAACAGACTAAGAA-3′. miRNAs targeted against human CPI-17 were based on siRNA sequences reported previously [6]: 5′-ACATTGATGAATTGTTGGAGTT -3′ (Hs1) and 5′-AACCTGTCGAGGACTTCATCCA-3′ (Hs2; cloned in tandem). miRNAs were cloned downstream of tGFP, a tGFP only vector was used as control. For miRNA-mediated double or triple knockdowns, miRNAs were cloned in tandem seperated by short spacer sequences on the same expression vector.

### Cell culture

NIH3T3 (immortalized Swiss mouse embryo fibroblasts) and HeLa (human cervix epitheloid carcinoma) cell lines were obtained from the European Collection of Animal Cell Cultures. RPM-MC (human recurrent primary melanoma) were a kind gift from Dr I Stamenkovic (MGH, Boston). M13, M18, M21 and M22 melanoma cell lines [16] were a kind gift from Keith S. Hoek, University Hospital of Zürich. Human primary melanocytes were obtained from Lonza. NIH3T3, HeLa and RPM-MC cells were grown in Dulbecco's modified Eagle Medium (4.5 g/L glucose, with L-glutamine, PAA Laboratories GmbH), supplemented with 10 % (vol/vol) donor calf serum (PAA Laboratories GmbH) for NIH3T3 or fetal bovine serum (PAA Laboratories GmbH) for HeLa and RPM-MC. Due to change in availability, DMEM, RPMI and FBS from Sigmal Aldrich was used for Figures 1D, 3A and 4A. M13, M18, M21 and M22 melanoma cell lines were grown in RPMI medium (with L-glutamin, Sigma Aldrich) supplemented with 1 mM sodium pyruvate (Life Technologies GmbH) and 10% (vol/vol) fetal bovine serum (Sigma Aldrich). Primary melanocytes were grown according to manufacturer's instructions (Lonza). All cells were maintained in standard cell culture conditions at 37°C and 5 % CO_2_.

### Plasmid and siRNA transfection

Plasmids and siRNA were transfected with Lipofectamine 2000 (Life Technologies GmbH), according to manufacturer's instructions. Stable clones of CPI-17 overexpressing cells were selected by treatment with 500 mg/ml G418 for 2 weeks and maintained thereafter in growth medium supplemented with G418 thereafter. Inducible overexpression was achieved by insertion of Ezrin-R579A cDNA into TetOn vector pUHD10.3 rtTA and expression was induced by Doxycyclin addition to the medium at a concentration of 1mg/ml.

### Lentiviral transduction

To generate stable knockdown cell lines, cells were transduced with lentiviral particles coding for tGFP and either shRNA under a U6 promoter or miRNA downstream of a CMV-driven tGFP. Virus particles were produced and concentrated as previously described [27]. Viral particles were concentrated 3-fold by polyethylene glycol precipitation (PEG-6000; Sigma) and used for overnight transduction of cells. For NIH3T3 cells, polybrene (Sigma) was added to a final concentration of 8 μg/ml. GFP-positive cells were selected by FACS.

### Expression of recombinant His-ezrin

Expression and purification of His-ezrin has been previously described [10]. In brief, *E. coli* cells (Rosetta 2 DE3; Novagen) were transformed with ezrin and grown in LB medium supplemented with antibiotics. Protein expression was induced for 4 h at 37°C by addition of 500 μM isopropyl β-d-1-thiogalactopyranoside (IPTG; Carl Roth GmbH) at an absorbance of 0.4-0.6 at 600 nm. Cells were collected by centrifugation, resuspended in a buffer containing 50 mM Tris (pH 8.0), 150 mM NaCl and protease inhibitors, before snap freezing in liquid nitrogen. After thawing, lysozyme (Carl Roth GmbH) was added and cells lysed by ultrasound. Lysates were cleared by centrifugation and incubated overnight with Ni-Sepharose 6 Fast Flow (GE Healthcare). After washing with buffer and buffer containing 30 mM imidazole, proteins were eluted with buffer containing 250 mM imidazole. Eluted protein was dialyzed against 50 mM Tris (pH 8.0) and 150 mM NaCl and stored at 4°C.

### Immunoprecipitation and pull-down assays

Immunoprecipitation was performed as previously described [28]. In brief, HeLa cells were lysed in 50 mM Tris (pH 7.4), 20 mM NaCl, 0.5% Lubrol 17A17, 1 mM sodium vanadate, 1 mM PMSF, 10 mg/mL aprotinin and 10 mg/mL leupeptin. Supernatants were incubated with 2 μg of antibody rotating for 1 h at 4°C. 30 μl protein A agarose was added and the mixture rotated for additional 3 h at 4°C. For pulldown of MYPT1, His-tagged Ezrin wildtype protein was coupled to Ni-Sepharose. HeLa cells were lysed in the same lysis buffer as for immunoprecipitation. Supernatant was incubated with His-Ezrin-Ni-Sepharose for 1 hour at 4°C. GTP-Ras pulldowns were performed using the Active Ras Pulldown and Detection Kit (Thermo Fisher Scientific), according to manufacturer's instructions. In brief, cells were lysed in 25mM Tris (pH 7.2), 150 mM NaCl, 5 mM MgCl2, 1% NP-40, 5% (vol/vol) glycerol, 1 x complete, EDTA-free protease inhibitor (Roche). Supernatants were incubated with GST-Raf1-RBD coupled to Glutathione agarose rotating for 1 h at 4°C. All sepharose/agarose complexes were washed 4× with cold lysis buffer, dissolved in 2× Laemmli sample buffer and subjected to SDS PAGE. Proteins were detected by Western Blot analysis.

### GEF activity assay

Intracellular GEF activity was measured as previously described [11]. In brief, NIH3T3 cells (vector control and CPI-17 overexpressing stable clones) were seeded in 6-well plates and grown to confluency. Cells were deprived of serum overnight, before being subjected to the GEF assay. Assay kinetics commenced after replacement of culture medium with 600 μl permeation buffer (50 mM HEPES pH 7.5, 107 mM K-glutamate, 23 mM NaCl, 3 mM MgCl_2_, 0.3 mM CaCl_2_, 1 mM EGTA, 1 mM ATP, 30 U/ml streptolysin O (Aalto Bioreagents), 100 mCi/ml [α^32^P]-GTP (Hartmann Analytik) and 10 ng/ml PDGF (except at 0 min)). At indicated time points, the exchange reaction was stopped by adding 1 ml lysis buffer (50 mM HEPES pH 7.5, 100 mM NaCl, 10 mM MgCl_2_, 1% NP-40, 100 mM GDP, 100 mM GTPγS, EDTA-free protease inhibitor (Roche) and 5 mg/ml anti-Ras antibody Y13-259). Lysates were cleared by centrifugation and supplemented to a final concentration of 500 mM NaCl, 0.6% sodium deoxycholate and 0.06% SDS. Ras was immunoprecipitated with protein G plus agarose (Oncogene). Precipitates were washed six times with 1 ml wash buffer (50 mM HEPES pH 7.5, 500 mM NaCl, 5 mM MgCl2, 0.1% Triton-X-100 and 0.005% SDS) and subjected to Cherenkov counting.

### Soft agar assay

For transformation, assays using soft agar cells were counted. 4 × 10^3^ cells were suspended in 1.8 ml medium and 200 μl agar was added to yield a final concentration of 0.33 % agar and 2 × 10^3^ cells/ml. 500 μl of this mixture were seeded in triplicates into 24-Well plates, immediately transferred to 4°C to ensure solidification of the agar and subsequently grown in a humidified cell culture incubator at 37°C and 5 % CO_2_ for two weeks. The resulting colonies, consisting of multiple cells, were counted. For M21 cells an updated protocol was used: 12-well plates were primed with an agarose bottom layer (800μl of 0.5% low-melt agarose in PBS), agarose was allowed to set at room temperature. Cells were mixed with low-melt agarose in complete medium at a final concentration of 1.25 × 10^4^ cells / ml, 10% FBS and 0.375% agarose respectively. 800 μl cell suspension (containing 1000 cells) was layered on top of the 0.5% bottom layer. Agarose was allowed to set at room temperature and a third layer of 800μl complete medium was added. Cells were incubated in a humidified cell culture incubator at 37°C and 5 % CO_2_ for 10 days, medium was refreshed once after 5 days. Cells were stained for 90 min with 0.005% Crystal Violett in distilled water and subsequently rinsed twice with destilled water. Wells were imaged using a Canon EOS 650D mounted on a Olympus SZX10 dissection microscope.

### Proliferation assay

Proliferation of M21 cells was measured using the Click-iT EdU Alexa 647 HCS assay (Thermo Fisher Scientific) following manufacturer's instructions. 5000 cells per well were seeded in a black, clear bottom 96 well plate. Six wells per cell line were seeded, cultivated overnight and then incubated with 10 μM EdU for 3 hours under standard cell culture conditions. Detection and quantification of EdU positive cells was performed using an Cellomics Arrayscan platform (Thermo Fischer Scientific). At least 1700 cells per well were detected by DAPI staining and EdU incorporation analyzed.

### Statistical analysis

Statistical significance was tested using two-tailed t-tests, F-tests were conducted to test the applicability of t-tests beforehand and accounting for differences in variance if neccessary. p-values of both tests are indicated within the respective figure legends.

### Microarray analysis

Expression of PPP1R14A mRNA coding for the protein CPI-17 was analyzed in different samples of primary human melanocytes and melanoma cells using the previously reported Zürich dataset [16]. The dataset was accessed at the NCBI GEO database [29], accession number GSE 4840, probe ID 227006_at. Signal intensities were normalized to the average of the three primary melanocyte samples, grouped by mutational status of BRAF and NRas (reported in Supplementary Data of [16]) and plotted in a bar chart (Figure 4A).

### Amplification and sequencing of BRAF and NRAS sequences

RPM-MC cells grown under standard conditions were trypsinized, washed with PBS and pelleted by centrifugation. Cell lysis was performed by addition of 0.025 NaOH, 0.2 mM EDTA, incubation at 98°C for 1h and neutralization by addition of 0.04 M Tris-HCl. Cell lysates were used as template for Q5 polymerase-mediated amplification of target sequences following manufactor's instructions. Primers for BRAF (forward 5′-GATTCTTACAGAAACAAGT-3′, reverse 5′-AACCTAAAACCAACTCTTCCCA-3′) and NRAS (forward 5′-AACACATTTCAAGCCCCAAA-3′, reverse 5′-AGCATCTCAGGGCCAAAAAT-3′) amplification were based on previous reports [30]. PCR reactions were seperated on an agarose gel and amplified DNA fragments isolated and purified using the Quiaquck Gelextraction kit (Qiagen) following manufactor's instructions. Isolated fragments were sequenced on an ABI sequencer 3730XL using the Big Dye Mix 3.0 according to manufacturer's instruction. Amplification primers were used for sequencing, as well. Sequencing information was aligned to the indicated reference sequences using the CLUSTAL OMEGA algorithm within the Benchling platform (www.benchling.com).

### Immunohistochemical stainings

The study was conducted according to the Declaration of Helsinki Principles and was supported by informed consent of the donors. Antigen expression was visualized by avidin-biotin-complex-technique using the Supersensitive Multilink alkaline Phosphatase Ready-to-use Detection system (Biogenix), according to manufacturer's instructions. In brief, paraffin sections were de-paraffinized by washing 3x in Xylol, 100% Ethanol, 96% Ethanol and finally 70 % Ethanol. Cold Methanol was used to unmask antigens. Samples were blocked by 2% goat serum in PBS. CPI-17 antibody was diluted 1:300 in blocking buffer and applied overnight at 4°C. Sections were washed twice for 5 min in PBS + 0.15% Tween20. Sections were incubated for 30 min with biotinylated anti-IgG, washed twice for 5 min in PBS + 0.15% Tween20, incubated for an additional 30 min with streptavidin-AP and washed again, twice, for 5 min in PBS/0.15% Tween20. Staining was performed using the New Fuchsin substrate system (Dako, Hamburg, Germany). Counterstaining was performed with hematoxylin.
