# Supplementary material for: CPI-17 drives oncogenic Ras signaling in human melanomas via Ezrin-Radixin-Moesin family proteins
Source: Oncotarget. 2016 Oct 26;7(48):78242–54. doi: 10.18632/oncotarget.12919 (PMC5346635; doi:10.18632/oncotarget.12919)
Supplement: Supplementary file 1 [file oncotarget-07-78242-s001.pdf]

CPI-17 drives oncogenic Ras signaling in human melanomas via Ezrin-Radixin-Moesin family proteins

Supplementary Material

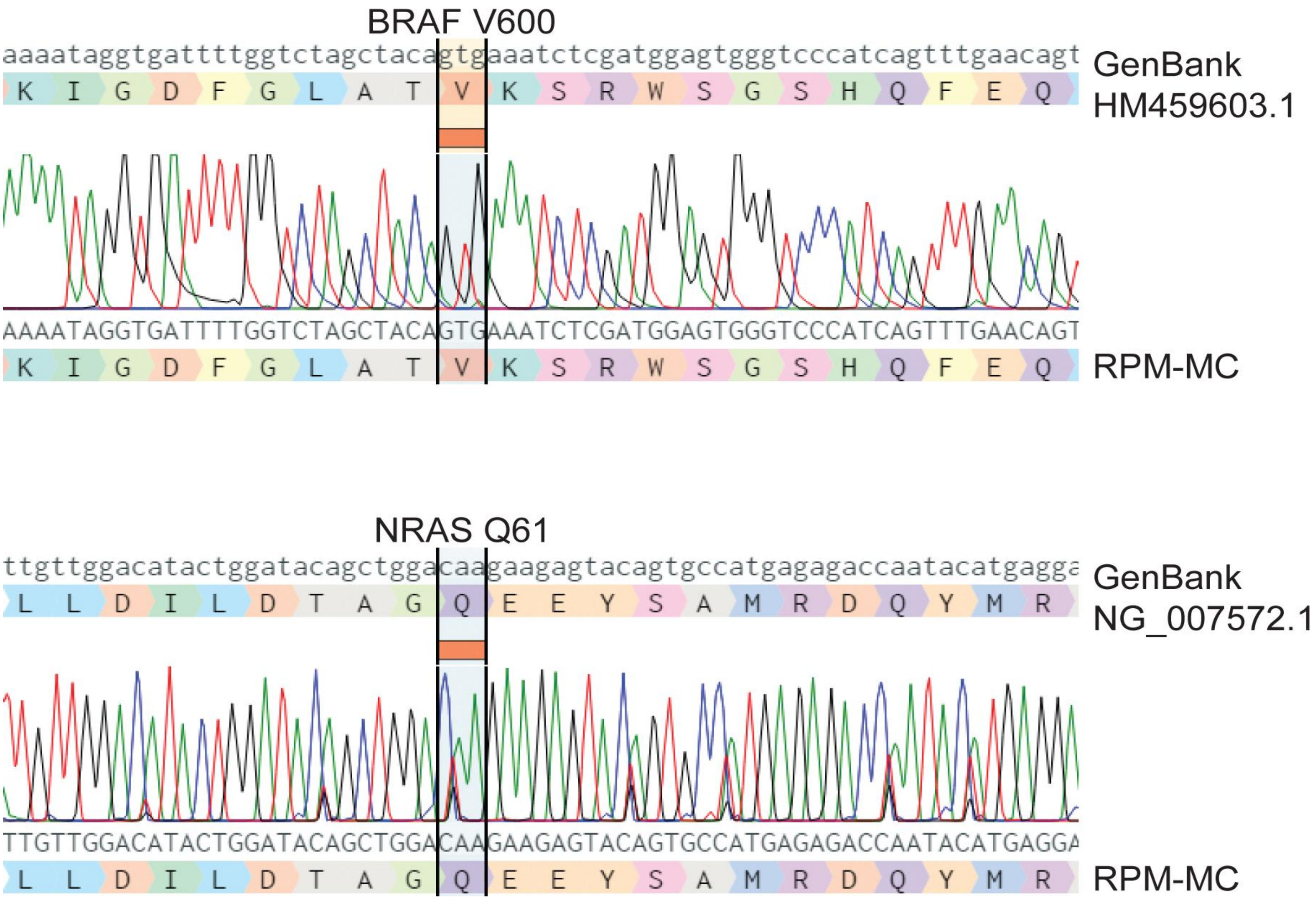

Figure S1. RPM-MC cells are wild type for BRAF V600 and NRAS Q61. DNA sequences from recurrent primary malignant melanoma cells (RPM-MC) surrounding the BRAF V600 and NRAS Q61 loci were amplified and sequenced. Alignment of sequencing results to indicated reference sequences deposited at NCBI GenBank was performed using the Clustal Omega algorithm within the Benchling online platform.

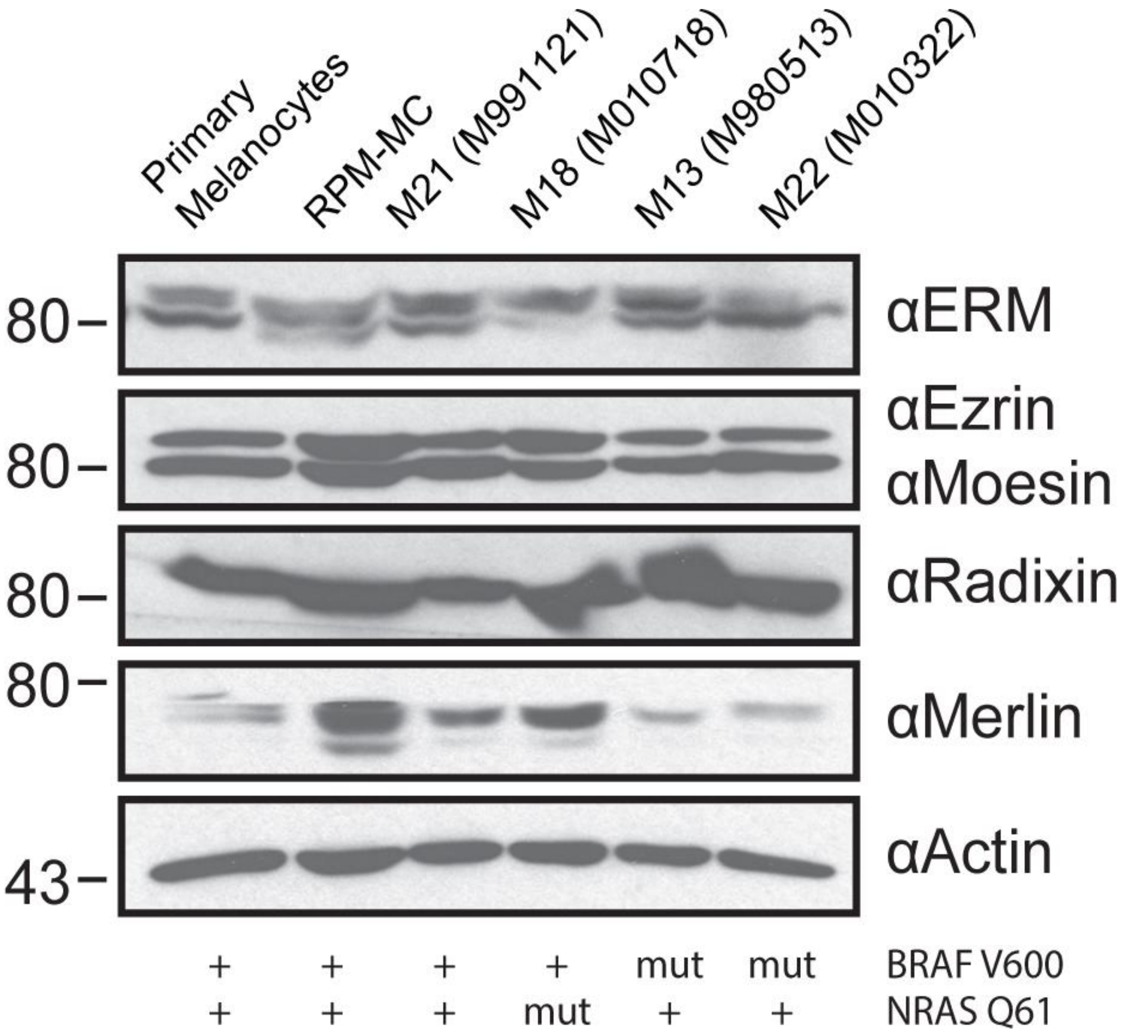

Figure S2. Expression of CPI-17 targets in primary human melanocytes and different patient-derived melanoma cell lines. Expression of CPI-17-activated ezrin, radixin and moesin (ERM) proteins appears to be unchanged. No loss of the CPI-17-inhibited tumor suppressor merlin was detected but rather appeared to be upregulated in three of five samples.

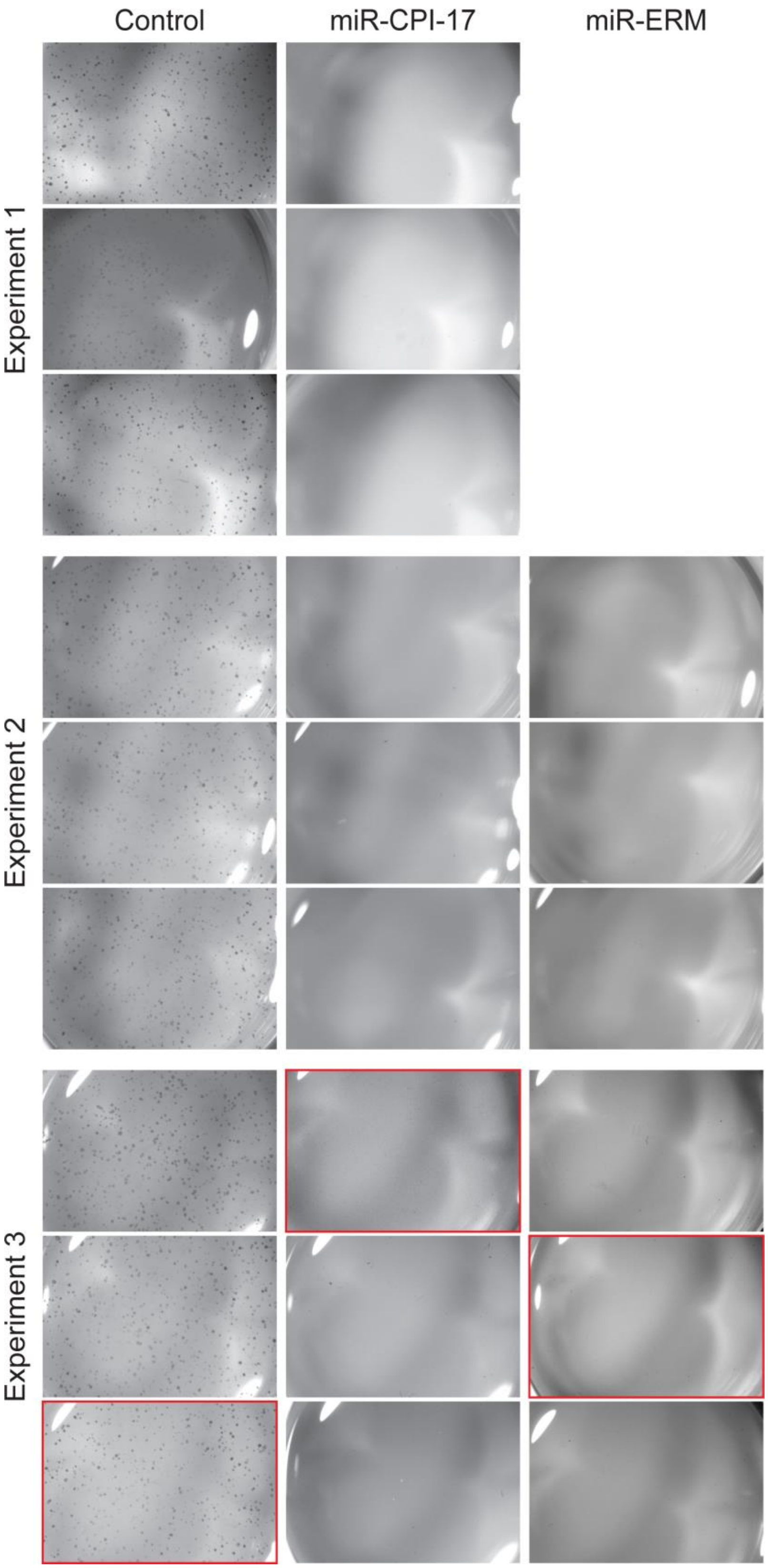

Figure S3. Depletion of either CPI-17 or ERM proteins in M21 cells decreases soft agar colony formation. 1 x 10^3 cells of respective cell lines were seeded in 12-well plates for a soft agar assay (see method sections) and grown for 10 days in complete medium. Cells were visualized by staining with 0.005% crystal violett. Pictures used as representative data in Figure 4D have been highlighted in red.
